# Insights Into Chemical Reactions at the Beginning of the Universe: From HeH^+^ to H_3_^+^

**DOI:** 10.3389/fchem.2021.679750

**Published:** 2021-06-18

**Authors:** Soumya Ranjan Dash, Tamal Das, Kumar Vanka

**Affiliations:** ^1^ Physical and Materials Chemistry Division, CSIR-National Chemical Laboratory (CSIR-NCL), Pune, India; ^2^ Academy of Scientific and Innovative Research (AcSIR), Ghaziabad, India

**Keywords:** density functional theory, ab initio molecular dynamics, ab initio nanoreactor, origin of molecules in the universe, the earliest lewis acid

## Abstract

At the dawn of the Universe, the ions of the light elements produced in the Big Bang nucleosynthesis recombined with each other. In our present study, we have tried to mimic the conditions in the early Universe to show how the recombination process would have led to the formation of the first ever formed diatomic species of the Universe: HeH^+^, as well as the subsequent processes that would have led to the formation of the simplest triatomic species: H_3_^+^. We have also studied some special cases: higher positive charge with fewer number of hydrogen atoms in a dense atmosphere, and the formation of unusual and interesting linear, dicationic He chains beginning from light elements He and H in a positively charged atmosphere. For all the simulations, the *ab initio* nanoreactor (*AINR*) dynamics method has been employed.

## Introduction

The way the Universe, and all the elements, came into being is one of the fascinating questions of science. Attempts to answer this question has led to the Big Bang theory, and an understanding of the primeval Universe and the entities that it was made up of (Meyer, 2008). Further advancement of science and technology has led to greater understanding, which led NASA’s Stratospheric Observatory for Infrared Astronomy (SOFIA) to the detection of HeH^+^ in the planetary nebula NGC 7027, the first molecule formed after the Big Bang (Güsten et al., 2019), 94 years after its discovery in the laboratory in 1925 (Hogness and Lunn, 1925).

**GRAPHICAL ABSTRACT:**
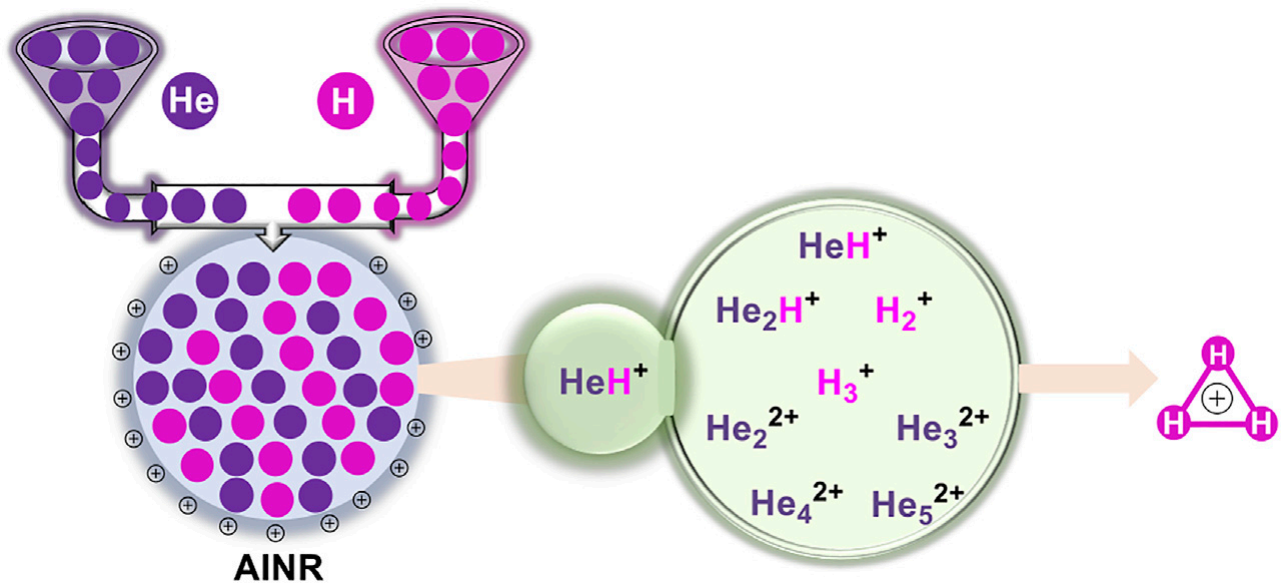


As the first molecule, the significance of the role of HeH^+^ in the evolution of other species cannot be overstated. One of these species, and perhaps the most important, is the simplest polyatomic molecule H_3_^+^, which has always intrigued researchers ever since its discovery in 1911 by J. J. Thomson (Sir Thomson, 1911). However, the importance H_3_^+^ in astrochemistry was realized only after it was detected on Jupiter in the 1980s (Oka, 1980; Drossart et al., 1989). High abundance of H_3_^+^ in the Universe and its ability to donate a proton established this triatomic cation as the interstellar acid of utmost importance for many extra-terrestrial reactions (Watson, 1973; Oka, 2013; Olah et al., 2016; Pelley, 2019). While there are many reports of H_3_^+^ formation from doubly ionized organic molecules (Townsend et al., 2004; De et al., 2006; Okino et al., 2006; Kushawaha and Bapat, 2008; Mebel and Bandrauk, 2008; Nakai et al., 2013; Ando et al., 2018; Ekanayake et al., 2018; Palaudoux and Hochlaf, 2019), our focus is on its origin and the role played by HeH^+^ on its formation.

H_3_^+^ formation was first reported to occur primarily from the combination of H_2_^+^ and H_2_, where H_2_^+^ would be formed from the ionization of H_2_ (Sir Thomson, 1912; Oka, 2013). There are other reports which state that H_2_^+^ is more likely to be formed from the combination of HeH^+^ and H (Bovino et al., 2011; Razio, 2014; Esposito et al., 2015; Fortenberry, 2019; González-Lezana et al., 2019). At the same time, the possibility of HeH^+^ combining with H_2_ to produce H_3_^+^ cannot be overlooked (McLaughlin and Thompson, 1973). Thus, many factors can influence the origin of H_3_^+^, but there have not been any conclusive studies yet.

In this work, we have employed the *ab initio* nanoreactor (AINR) method to carry out full quantum mechanical molecular dynamics (MD) simulations on systems containing atoms/ions of helium and hydrogen, and have obtained reaction profiles by varying their mixture ratio and the charge. The *AINR* method, developed by Martinez and co-workers, allows the determination of new reaction pathways and products, without the need of controlling the chemical system (Zimmerman, 2013; Rappoport et al., 2014; Wang et al., 2014). Our primary goal was to gain insight into the formation of different species from the combination of He and H in the presence of a positively charged atmosphere, as well as their further dissociation and recombination. As the Results and Discussion section will show, our studies provide interesting new insights into HeH^+^ formation, and shed light on various short-lived intermediates that could have formed *en route* to obtaining H_3_^+^– the stable species that was known to exist in the early Universe (Oka, 2013).

## Computational Methods

### *Ab Initio* Molecular Dynamics Simulations

The AIMD simulations were performed with the TeraChem 1.9 (Ufimtsev and Martínez, 2008a; Ufimtsev and Martínez, 2008b; Ufimtsev and Martinez, 2009a; Ufimtsev and Martinez, 2009b; Ufimtsev et al., 2011; Isborn et al., 2011; Titov et al., 2013) software package using the Hartree−Fock (HF) (Fischer, 1987) electronic wave function and the 6–311 g (Binkley et al., 1980) Gaussian basis set, to calculate the Born−Oppenheimer potential energy surface. This method has been implemented in TeraChem by Martinez and co-workers. This approach was deemed acceptable because the HF method is well-known for predicting chemically reasonable structures (Feller and Peterson, 1998). Also, it should be noted that HF was not employed to determine the thermodynamics of the reactions: its only role was in the discovery process. This was also the approach employed by Martinez and co-workers in their original *AINR* paper (employing HF/3–21 g), where they replicated the results obtained from the Urey−Miller experiment, as well as from the interaction of acetylene molecules. The same method (HF) was also employed by us in our previous report (Das et al., 2019) on reaction pathways leading to the formation of precursors of RNA and sugars. Electrostatic interactions were treated using two of the most common methods: the residue-based cutoff and particle mesh Ewald (PME). The PME method has been chosen because it takes care of long-range electrostatic interactions and is the most widely used approach.

The results were obtained from the *AINR* simulations by varying both the He to H ratio, as well as the positive charge of the system. Each simulation was repeated thrice. The system was constrained in a spherical boundary of 4.0 and 2.0 Å radii, so that the atoms resided in a space that alternated between the volumes created by these two radii, and collided with each other. Each *AINR* dynamics was evolved upto 15 ps, with a time step of 0.5 fs.

Newton’s equations of motion were calculated using Langevin dynamics, with an equilibrium temperature of 1,000.0 K (also the starting temperature of the dynamics). We have used this high temperature in order to increase the average kinetic energy of the reactants and for faster dynamics (Novotný et al., 2019). We have employed the augmented direct inversion in the iterative subspace (ADIIS) algorithm (Hu and Yang, 2010) available in TeraChem as an alternative tool for self-consistent field calculations at each AIMD step in which the default DIIS algorithm (Pulay, 1980) failed to converge. The nanoreactor simulations employ a virtual piston by fluctuating the radius of a spherical boundary, which allows the continuous expansion and compression of the system, thus artificially changing the pressure and the density for the collision cycles, which is necessary for the molecules to collide and also increases the rate of the reactions. Spherical boundary conditions (details in the ESI) were applied to prevent the molecules from flying away, a phenomenon known as the “evaporation” event. For more details, please check the original paper on the *AINR* by Martinez and co-workers (Wang et al., 2014).

The mechanistic pathways obtained from the *AINR* simulations were then analyzed with full quantum mechanical (QM) calculations. All the structures were optimized with coupled cluster singles doubles (CCSD) (Grotendorst et al., 2006) and with the 6–311++G (d,*p*) (McLean and Chandler, 1980) basis set. The Gaussian09 software (Frisch et al., 2009) was employed for the thermodynamic calculations. The complete solution of the H_3_^+^ problem requires the consideration of relativistic effects, nuclear motion, and breakdown of the Born-Oppenheimer (B-O) approximation (both adiabatic and non-adiabatic) (Miller et al., 2020). Recent studies have shown that relativistic effects for H_3_^+^ are quite negligible (Cencek et al., 1998; Bachorz et al., 2009). Issues of breakdown of the B-O approximation become relevant when considering transition frequencies of H_3_^+^, i.e., in spectroscopic studies. Since the current work is focused on understanding the possible chemical reactivity and thermochemistry of HeH^+^ and H^+^, leading eventually to H_3_^+^ formation, the current studies, done within the B-O approximation, are appropriate.

## Results and Discussions

In this section, we will briefly describe the formation of H_3_^+^ in the *AINR via* different short lived intermediates. We have taken a fixed composition of the He and H mixture and varied the overall positive charge density of the system (as shown in Tables 1, 2). During the simulations in each case, it was seen that HeH^+^ was formed at the very beginning of the dynamics as the first molecular species. In our first set of simulations, we have taken a homogeneous mixture of 30 atoms each of H and He. The *AINR* makes them collide with each other at a temperature of 1,000.0 K. The simulation with no positive charge in the system does not produce any intermediates and H_3_^+^ at all throughout the dynamics. This led us to consider the possibility that a more appropriate set-up would include a positively charged system, which would mimic the collisions between the ionized state of the helium and hydrogen atoms present at the beginning of Universe (Oka, 2013). A positively charged environment for the formation of H_3_^+^ had also been considered by many previous reports, while investigating its origin from different organic molecules (Pilling et al., 2007). Therefore, we have varied the positive charge of the system by even numbers (Table 1) during the *AINR* dynamics. As the dynamics progressed, various short lived species such as He_2_^2+^, He_3_^2+^ and He_2_H^+^ (snapshots shown in the Supplementary Figures S1–S5 in the Supporting Information (ESI) file) were seen to have formed in almost every simulation, though their time of appearance was different in each case. It was also observed that with the increase of the positive charge of the system, the formation of H_3_^+^ ions also increased, up to a point. The number of H_3_^+^ ions generated was equal to the positive charge in the system, up to a charge of +6 (see Table 1).

**TABLE 1:** *AINR* simulations with 30 He atoms and 30 H atoms: different entries represent the variation of the total positive charge of the system–by even numbers.

Total charge	First molecule	Intermediate species	Dominant end molecule	No. of H_3_^+^
0	-	-	-	-
2	HeH^+^	He_2_^2+^, He_3_^2+^, He_2_H^+^, H^+^, H_2_	H_3_^+^	2
4	HeH^+^	He_2_^2+^, He_2_H^+^, H^+^, H_2_	H_3_^+^	4
6	HeH^+^	He_2_^2+^, He_3_^2+^, He_2_H^+^, H^+^, H_2_	H_3_^+^	6
8	HeH^+^	He_2_^2+^, He_3_^2+^, He_2_H^+^, H^+^, H_2_	H_3_^+^	7
10	HeH^+^	He_2_^2+^, He_3_^2+^, He_2_H^+^, H^+^, H_2_	H_3_^+^	7
20	HeH^+^	He_2_^2+^, He_2_H^+^, H^+^, H_2_	H_3_^+^	5

**TABLE 2:** *AINR* simulations with 30 He atoms and 29 H atoms: different entries represent the variation of the total positive charge of the system–by odd numbers.

Total charge	First molecule	Intermediate species	Dominant end molecule	No. of H_3_^+^
0	-	-	-	-
1	HeH^+^	He_2_H^+^, H^+^, H_2_	H_3_^+^	1
3	HeH^+^	He_2_^2+^, He_2_H^+^, H^+^, H_2_	H_3_^+^	3
5	HeH^+^	He_2_^2+^, He_2_H^+^, H^+^, H_2_	H_3_^+^	5
7	HeH^+^	He_2_^2+^, He_3_^2+^, He_2_H^+^, H^+^, H_2_	H_3_^+^	6
9	HeH^+^	He_2_^2+^, He_3_^2+^, He_2_H^+^, H^+^, H_2_	H_3_^+^	7
11	HeH^+^	He_2_^2+^, He_2_H^+^, H^+^, H_2_	H_3_^+^	7
21	HeH^+^	He_2_^2+^, He_2_H^+^, H^+^, H_2_	H_3_^+^, HeH^+^	4

However, upon further increase in the positive charge of the system beyond six–to eight or ten, the number of H_3_^+^ ions formed was not seen to be equal to the total positive charge of the system. Instead of H_3_^+^, the remaining positive charge of the system was balanced by H^+^ or, in some cases, HeH^+^. As shown in Table 1, in case of a positive charge of 10 and after 250 fs, we observed only seven H_3_^+^ ions remaining with three H^+^, which balanced the total charge of the system. The natural population analysis (NPA), or the formal charge analyzed data for all the atoms in several snapshots, has been shown in Supplementary Tables S1–S4 in the ESI.

Similarly, in another set of MD simulations, we have taken 29 H with 30 He atoms and varied the overall charge of the system by an odd number: one, three, five and so on. These observations have been shown in Table 2. We have observed a similar trend for the formation of H_3_^+^ as the only end product up to a certain limit (here, the value is 5) of positive charge and beyond that, the total charge of the system was seen to be balanced by the sum of H_3_^+^, H^+^ and HeH^+^, as seen in the previous section when the positive charge was varied by even numbers.

In short, we can say that in all the cases of AINR dynamics studied, the formation of HeH^+^ as the first molecule was observed. However, upon varying the total positive charge of the whole system, several short-lived species (He_2_H^+^, He_3_^2+^, He_2_^2+^) were observed (Tables 1, 2) after HeH^+^ formation. At the end of the simulation, H_3_^+^ and H_2_ were found to be the only stable species left in the reaction mixture.

### The Timescale of Formation of H_3_^+^ and Other Short Lived Molecules

**FIGURE 1:**
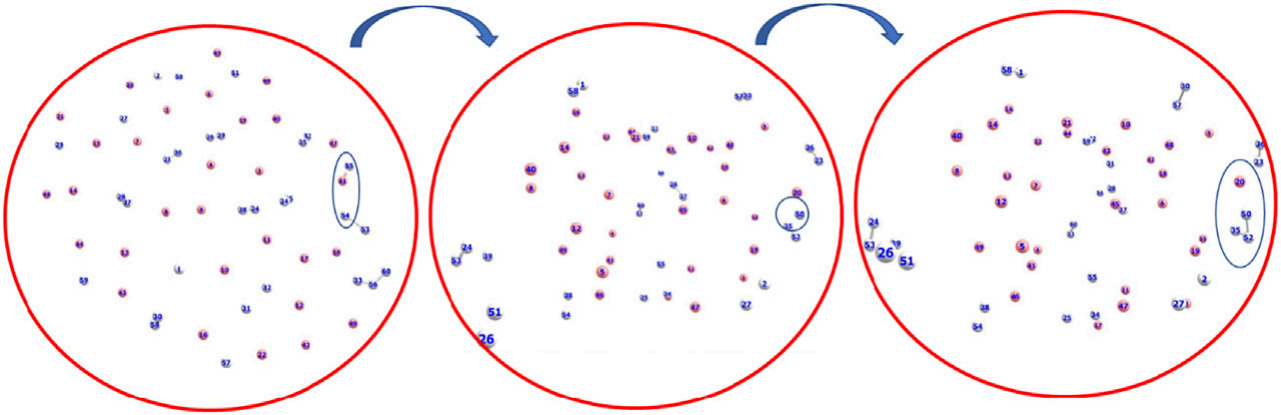
Snapshots of *AINR* simulations showing the progress of the dynamics starting from atomic He and H, leading toward the formation of H_3_^+^ from HeH^+^ and dihydrogen. HeH^+^ was seen to be formed very early–near the beginning of the dynamics (Color: He - peach, H - white).

The formation timescale of different short lived species, along with the stable H_3_^+^, has been observed from femtosecond *AINR* simulations. In each and every simulation, HeH^+^, which has been proposed to be the first formed molecule, was seen to be formed soon after the beginning of the dynamics. The time of appearance of HeH^+^ was within 15 fs timesteps. Subsequently, other short lived species (He_2_H^+^, He_3_^2+^, He_2_^2+^) were formed within the timescale of 0.1 ps (shown in Table 3). The observed timescale for the existence of such transient species is around 5–10 fs. Once these molecules were formed, they quickly dissociated and this ultimately led to the formation of H_3_^+^, which was observed for every case. From the AINR dynamics, we have analyzed the data and found two pathways for the formation of H_3_^+^, starting from He and H in atomic states within the positively charge atmosphere. Both of the pathways involved the well-known roaming hydrogen mechanism (Townsend et al., 2004; Nakai et al., 2013; Palaudoux and Hochlaf, 2019). An mp4 file (Supplementary Movie S1) of a movie made of a part of an *AINR* simulation is included in the ESI. The most feasible pathway for H_3_^+^ formation is the abstraction of a proton from the first molecule HeH^+^ by the roaming dihydrogen (shown in Figure 1).

**TABLE 3:** Time (in fs) of the first appearance of different species.

Total charge	HeH^+^	He_2_H^+^	He_2_^2+^	He_3_^2+^	H_3_^+^
0	-	-	-	-	-
2	4.5	11.0	10.0	25.5	27.0
4	9.0	25.5	19.5	80.0	45.0
6	5.0	12.5	16.0	49.5	9.0
8	4.0	12.5	22.5	53.0	8.0
10	5.5	18.0	14.0	19.0	7.0
20	4.0	26.5	14.5	22.0	9.0

**SCHEME 1:**
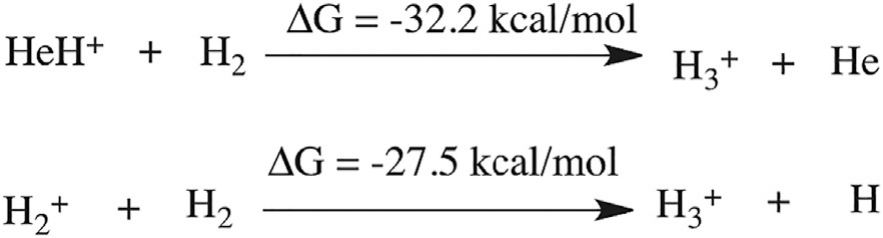
Thermodynamics of H_3_^+^ formation.

The thermodynamics for this step has been calculated to be −32.2 kcal/mol (shown in Scheme 1). In another mechanistic pathway, there is no involvement of HeH^+^. Instead of HeH^+^, the proton abstraction occurs from a mono-cationic dihydrogen molecule by the roaming dihydrogen. This process is thermodynamically favourable by 27.5 kcal/mol.

The total number of H_3_^+^ molecules formed was also found to be directly correlated with the total charge of the system, as well as the number of He and H atoms taken. Greater charge in the system yielded more short-lived species during the simulations. Most of the intermediate species were found to be formed within 100 fs (Table 3) and they were found to exist for only about 5–10 fs during the *AINR* simulations.

Since it has been postulated that different ratios of helium to hydrogen atoms could have existed in the early Universe (Meyer, 2008), we have further performed *AINR* dynamics with a 1:3 ratio of helium to hydrogen atoms and varied the total positive charge of the system (see Table 4). Furthermore, in order to investigate the effect of temperature on cosmic reionization (Novotný et al., 2019), we have also carried out *AINR* simulations while varying the temperature (Table 5), with a 1:3 ratio of He:H and a fixed positive charge (8^+^). In all such simulations, we have observed trends similar to those discussed in the previous sections, like the formation of HeH^+^ as the first molecule and the subsequent formation of transient species (He_2_H^+^, He_3_^2+^, He_2_^2+^), leading eventually to H_3_^+^ formation.

**TABLE 4:** Time of occurrence (in fs) of different species from the *AINR* simulation of 1: 3 ratio of helium to hydrogen while varying total positive charge of the system.

Total charge	HeH^+^	He_2_H^+^	He_2_^2+^	He_3_^2+^	H_3_^+^
0	-	-	-	-	-
4	5.0	74.5	14.0	24.0	12.5
6	9.5	15.5	12.0	27.0	17.0
8	6.0	54.0	15.0	19.5	21.5
10	10.0	16.0	14.5	30.0	19.0
12	6.5	70.5	16.0	23.0	23.0

**TABLE 5:** Time of occurrence (in fs) of different species from the *AINR* simulation of a 1: 3 ratio of helium to hydrogen while varying the temperature, with a fixed total positive charge of system (8^+^).

Temperature (K)	HeH^+^	He_2_H^+^	He_2_^2+^	He_3_^2+^	H_3_^+^
3,300	3.0	24.5	10.0	26.0	5.5
2,500	8.5	27.0	14.0	18.0	63.0
2,000	7.0	22.0	16.0	19.0	25.0
1,500	7.0	14.0	11.5	17.0	60.0
1,000	6.0	54.0	15.0	19.5	21.5
500	8.0	16.0	15.0	20.5	47.0

**FIGURE 2:**
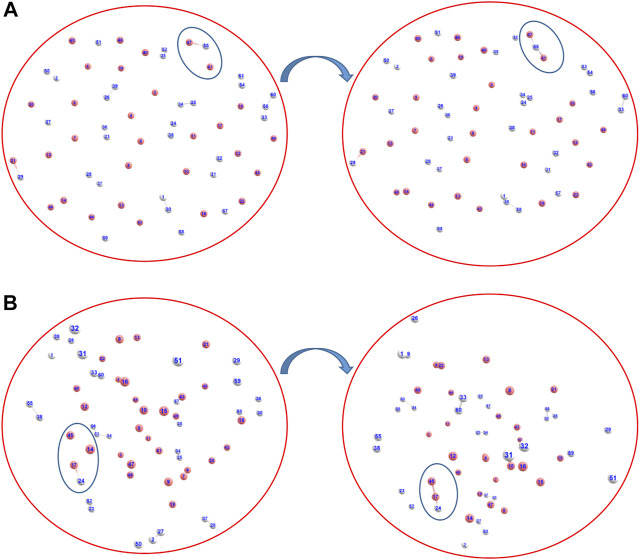
Snapshots of *AINR* simulations revealing the pathway toward the making of He_2_H^+^, in the form of **(A)** [He-H-He]^+^ and **(B)** [He-He-H]^+^ (Color: He - peach, H - white).

We have also addressed the speculation on the exact nature of the formed ion He_2_H^+^: whether it was formed as [He-H-He]^+^ or as [He-He-H]^+^ (Kim and Lee, 1999), *via AINR* dynamics followed by static CCSD calculations. As shown in Figures 2A,B, two different routes leading to the formation of [He-H-He]^+^ and [He-He-H]^+^ were observed during the simulations, generated from the collision of HeH^+^ and He. The thermodynamics was evaluated and it was found that the formation of the [He-H-He]^+^ species was exergonic by 32.3 kcal/mol, whereas the formation of [He-He-H]^+^ was only favourable by 4.5 kcal/mol. In other words, our calculations indicate that He_2_H^+^ would have formed predominantly as [He-H-He]^+^ rather than [He-He-H]^+^.

In another set of simulations, we have taken a different ratio of helium to dihydrogen and simultaneously varied the total charge of the system. In these cases, due to the high charge density, the dihydrogen quickly dissociated into a proton and atomic hydrogen. Here too, we have observed similar trends: 1) HeH^+^ is the first molecule to be formed, followed by 2) the formation of other short lived species, leading to H_3_^+^, which remained at the end, along with one or two molecules of HeH^+^ (shown in Table 5). For the case of 20 He and five H_2_ having a total of eight positive charge in the system, for instance, we observed that after a few collisions, there was still one HeH^+^ molecule present along with one H_3_^+^ and that they were in equilibrium with each other, due to the instantaneous proton transfer between HeH^+^ and H_2_. Similar trends were observed for other simulations where the total positive charge of the system was high (in our simulation conditions, the values were ≥16). It is worth mentioning that in this high positive charge atmosphere with comparatively low H atom density, the number of H_3_^+^ that survived after the collisions was either one or two, depending upon the ratio of He to H_2_ (shown in Table 6). Also, due to the very high positive charge density and high temperature (1,000.0 K) the movement of the light H^+^ ions was seen to be extremely fast and they repelled each other, going far away. This reduced the propensity toward the formation of H_3_^+^ in such simulations.

**TABLE 6:** Different ratios of He to dihydrogen while varying the total positive charge of the system.

No. of He	No. of H_2_	Total charge	First molecule	Intermediate species	Dominating end molecule	No. of H_3_^+^
20	5	8	HeH^+^	He_2_^2+^, H^+^	HeH^+^, H_3_^+^	1
30	10	16	HeH^+^	He_2_^2+^, He_3_^2+^, He_2_H^+^, H^+^	HeH^+^, H_3_^+^	1
30	15	24	HeH^+^	He_2_^2+^, He_2_H^+^, H^+^	HeH^+^, H_3_^+^	1
30	15	26	HeH^+^	He_2_^2+^, He_2_H^+^, H^+^	HeH^+^, H_3_^+^	1
15	10	8	HeH^+^	He_2_^2+^, He_2_H^+^, H^+^	H_3_^+^	4
30	15	20	HeH^+^	He_2_^2+^, He_3_^2+^, He_2_H^+^, H^+^	HeH^+^, H_3_^+^	4

### Formation of Unique Dicationic He Chains

**FIGURE 3:**
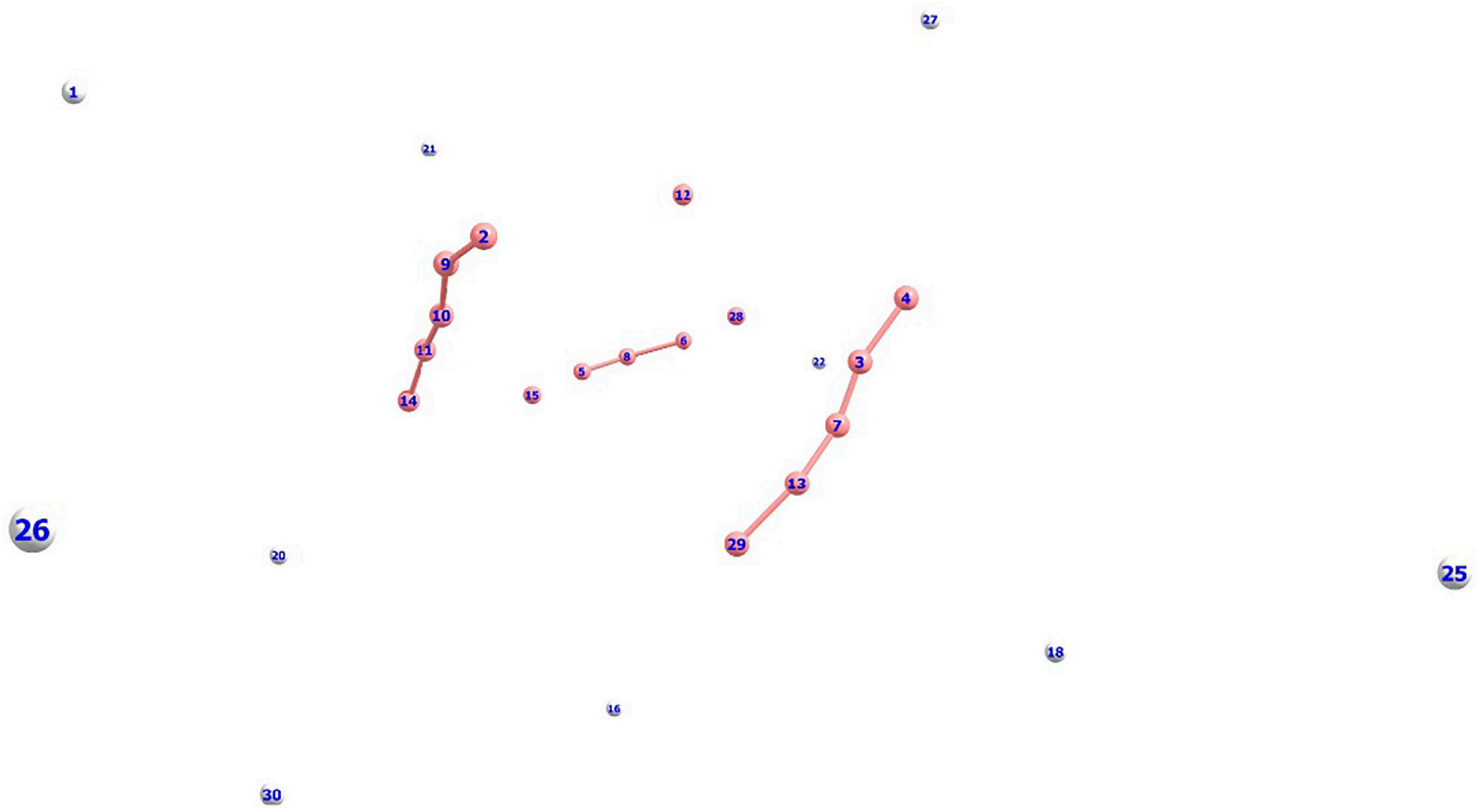
Dicationic He chain formation during an *AINR* simulation of 15 H and 15 He atoms, with an overall positive charge of 20 (Color: He - peach, H - white).

Previously, there have been some reports (Marinetti et al., 2008; Oleksy et al., 2010) with regard to the formation of mono-cationic He ion clusters. Our current *AINR* based dynamics study reveals that there is a possibility of the formation of a dicationic helium chain of up to five He atoms: He_3_^2+^, He_4_^2+^ and He_5_^2+^. This interesting result was obtained when we took a homogeneous mixture of H and He (15 atoms each) in the AINR, with an overall positive charge of 20 for the system. After a certain amount of time had elapsed (1 ps), we observed that a chain like structure had formed comprising of up to five helium atoms (shown in Figure 3).

**SCHEME 2:**
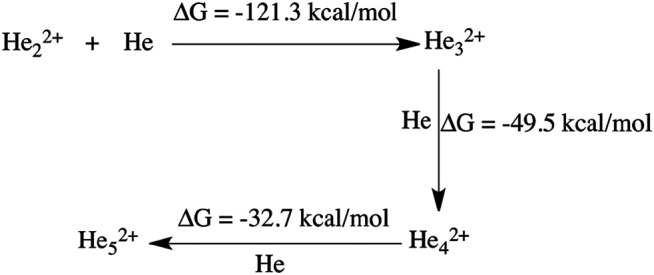
Thermodynamics of helium chain formation (up to He_5_^2+^).

An mp4 file (Supplementary Movie S2) of a movie made of a part of such an *AINR* simulation is included in the ESI. We have taken snapshots during the dynamics and carried out natural population analysis (NPA), in order to calculate the charge on the He atoms in the formed linear chain. From the NPA charge analysis (shown in Supplementay Table S6 in the ESI), it has been confirmed that all the formed He chains (He_3_^2+^, He_4_^2+^, He_5_^2+^) were dicationic in nature. For further confirmation of the stability of these dicationic He chains, we have done thermodynamics calculations for the formation of the He chain starting from He_2_^2+^ (shown in Scheme 2). The Gibbs free energy values suggest that the formation of the dicationic helium chain up to He_5_^2+^ is favourable, but further formation of He_6_^2+^ is thermodynamically not feasible. For this reason, we did not observe any He chain beyond five He atoms in our *AINR* simulations.

## Conclusion

In our current work, we have tried to shed light on the chemical reactions that might have taken place at the beginning of the Universe. We have focused on how, at the very beginning, simple molecules came into being after the Big Bang. We have investigated how He and H atoms, which were the first atoms formed, collided with each other in a positively charged atmosphere. This has been done by using a fresh computational approach–by employing the *ab initio* nanoreactor (*AINR*). The simulations reveal the presence of unique dicationic helium chains of up to five atoms, which should act as a fillip for investigating the possibility of the presence of such species in helium clusters, which have received attention both from experimental and theoretical studies (Bieske and Dopfer, 2000; Marinetti et al., 2008; Oleksy et al., 2010). Our studies also confirm that HeH^+^ was indeed the first molecule to be formed and that it played a vital role in the origin of H_3_^+^. The preservation of H_3_^+^, as a relatively stable species, in each of the simulations after every collision cycle, also explains the high abundance of H_3_^+^ in the early Universe. As such, our work provides interesting computational insights into the origin of unique and interesting molecules at the dawn of the Universe.

## Data Availability

The original contributions presented in the study are included in the article/Supplementary Material, further inquiries can be directed to the corresponding author.

## Abbreviations

AIMD, *Ab initio* molecular dynamics; AINR, *Ab initio* nano reactor; DFT, Density functional theory.
