# Supplementary material for: Insights Into Chemical Reactions at the Beginning of the Universe: From HeH+ to H3 +
Source: Front Chem. 2021 Jun 18;9:679750. doi: 10.3389/fchem.2021.679750 (PMC8249737; doi:10.3389/fchem.2021.679750)
Supplement: Supplementary file 3 [file DataSheet1.docx]

**Supporting Information**

**Insights into Chemical Reactions at the Beginning of the Universe: From HeH^+^ to H_3_^+^**

Soumya Ranjan Dash, Tamal Das and Kumar Vanka^*^

*Email: k.vanka@ncl.res.in

**Table of Contents**

AINR Spherical Boundary Conditions

**Figure S1**. Snapshot of H_3_^+^ formation during the dynamics with 30 H and 30 He atoms with the aid of the *AINR* approach.

**Table S1.** NPA charge analysis during the formation of H_3_^+^ at the M06-2X/6-311++g level of theory for the *AINR* dynamics with 30 atoms of H and 30 atoms of He taking six overall positive charge of the system.

**Figure S2**. Snapshot of HeH^+^ formation during the dynamics with 30 H and 30 He atoms with the aid of *AINR* approach.

**Table S2.** NPA charge analysis during the formation of HeH^+^ at the M06-2X/6-311++g level of theory for the *AINR* dynamics with 30 atoms of H and 30 atoms of He taking six overall positive charge of the system.

**Figure S3.** Snapshot of formation of short-lived species He_2_H^+^ during the dynamics with 30 H and 30 He atoms with the aid of the *AINR* approach.

**Table S3.** NPA charge analysis during the formation of He_2_H^+^ at the M06-2X/6-311++g level of theory for the *AINR* dynamics with 30 atoms of H and 30 atoms of He taking six overall positive charge of the system.

**Figure S4.** Snapshot of formation of short lived species He_3_^2+^ during the dynamics with 30 H and 30 He atoms with the aid of the *AINR* approach.

**Table S4.** NPA charge analysis during the formation of He_2_^2+^ at the M06-2X/6-311++g level of theory for the *AINR* dynamics with 30 atoms of H and 30 atoms of He taking six overall positive charge of the system.

**Figure S5**. Dicationic He chain formation during the *AINR* simulation of 15 H and 15 He with overall 20 positive charge.

**Figure S6.** Dicationic He chain formation during the *AINR* simulation of 15 H and 15 He with overall 20 positive charge.

**Table S5**. NPA charge analysis at the M06-2X/6-311++g level of theory for the AINR dynamics with 15 atoms of H and 15 atoms of He taking 20 overall positive charge of the system.

**Figure S7**. Formation of dicationic He_5_ chain during the dynamics with 15 H and 15 He atoms taking twenty overall positive charge with the aid of the *AINR* approach.

**Table S6**. NPA charge analysis by M06-2X/6-311++g level of theory for the AINR dynamics with 15 atoms of H and 15 atoms of He taking 20 overall positive charge of the system (as shown in Figure S7).

**Movie S1.** The *ab initio* nanoreactor dynamics simulation movie starting from atomic He and H and leading to the formation of H_3_^+^ *via* different short-lived intermediate species.

**Movie S2.** The *ab initio* nanoreactor dynamics simulation movie for dicationic He chain formation.

The Cartesian (x,y,z) Coordinates of all the starting *AINR* geometries at the HF/6-311g level of theory, obtained with the TeraChem software.

**AINR Spherical Boundary Conditions:**

Spherical boundary conditions were applied to prevent the molecules from flying away, a phenomenon known as the “evaporation” event. The spherical boundary conditions were provided in the form of a sum of two harmonic terms. The molecules were restricted to move inside a spherical volume by a boundary potential, with a time-dependent component:

V(r, t)= f(t)U(r,r_1_,k_1_) +(1− f(t)) U(r,r_2_,k_2_)

U(r,r_0_,k) = mk/2 (r – r_0_)^2^θ(r – r_0_); f (t) = θ(⌊ t/T ⌋ − t/T + τ/T)

Where k_1_ =2.0 kcalmol-1Å-^2^, r_1_ =4.0 Å, k_2_ =1.0 kcalmol-^1^Å-^2^, r_2_ =2.0 Å, ⌊⌋ is the floor function and θ is the heaviside step function. The function f(t) is a rectangular wave that oscillates between one (duration τ) and zero (duration T–τ), and U(r, r_0_, k) is a radial potential that is zero inside the prescribed radius r_0_ and harmonic outside. The force constant is multiplied by the atomic mass (in a.m.u) such that all the atoms at the same radial coordinate have attained equal acceleration. The rectangular waveform switches the restraint potential between U(r, r_1_, k_1_) and U(r, r_2_, k_2_), which forces the atoms with a radial position 4.0 Å to 2.0 Å towards the centre of the sphere and allows them to collide. When the sphere is expanded again, the molecules present in the smaller volume diffuse rapidly (because of the high simulation temperature, here it is 1000 K) to occupy the larger volume. Due to the repeating compression and expansion of spherical volume, the molecules collide and relax. Therefore, throughout the simulation, new molecules are formed and then break again to form other new molecules.

|  |
| --- |

**Figure S1**. Snapshot of H_3_^+^ formation during the dynamics with 30 H and 30 He atoms, with the aid of the *AINR* approach.

**Table S1**. NPA charge analysis during the formation of H_3_^+^ at the M06-2X/6-311++g level of theory for *AINR* dynamics with 30 atoms of H and 30 atoms of He taking six overall positive charge of the system (as shown in Figure S1).

1 H 0.573382

2 H 0.316347

3 He 0.000049

4 He 0.001070

5 He -0.025062

6 He 0.017875

7 He -0.012695

8 He 0.002093

9 He -0.000391

10 He 0.000857

11 He -0.022286

12 He 0.035954

13 He -0.003533

14 He 0.002714

15 He 0.011648

16 He -0.000715

17 He 0.033993

18 He 0.007595

19 He 0.005249

20 He 0.007972

21 He 0.002421

22 He 0.009109

23 H 0.229751

24 H 0.554960

25 H 0.054350

26 H -0.016488

27 H 0.120264

28 H 0.485546

29 H 0.530196

30 H -0.025162

31 H -0.153561

32 H 0.100482

33 H 0.067169

34 H -0.083691

35 H 0.240220

36 H 0.073855

37 H 0.054602

38 H -0.011588

39 H 0.010768

40 He 0.018407

41 He -0.001652

42 He 0.012434

43 He 0.002044

44 He -0.004242

45 He 0.055356

46 He 0.003929

47 He 0.003089

48 He 0.008388

49 He 0.002616

50 H 0.031117

51 H 0.438391

52 H 0.117793

53 H 0.367704

54 H 0.525372

55 H 0.050342

56 H 0.048642

57 H 0.532769

58 H 0.120013

59 H 0.530950

60 H -0.058782

|  |
| --- |

**Figure S2**. Snapshot of HeH^+^ formation during the dynamics with 30 H and 30 He atoms with the aid of the *AINR* approach.

**Table S2.** NPA charge analysis during the formation of HeH^+^ at the M06-2X/6-311++g level of theory for A*INR* dynamics with 30 atoms of H and 30 atoms of He taking six overall positive charge of the system (as shown in Figure S2).

1 H 0.150378

2 H 0.103890

3 He 0.030261

4 He 0.047507

5 He 0.000395

6 He 0.010223

7 He 0.033169

8 He 0.020555

9 He 0.055115

10 He 0.040405

11 He 0.063158

12 He 0.047837

13 He -0.006833

14 He 0.017698

15 He 0.005365

16 He 0.041760

17 He 0.007567

18 He 0.076067

19 He 0.026108

20 He 0.091886

21 He 0.309189

22 He 0.169717

23 H 0.044567

24 H -0.095912

25 H -0.081616

26 H 0.066592

27 H 0.184662

28 H -0.085639

29 H 0.678167

30 H -0.144519

31 H -0.107902

32 H 0.103264

33 H 0.296087

34 H 0.182420

35 H -0.124380

36 H 0.002785

37 H 0.087846

38 H -0.103307

39 H -0.214683

40 He 0.033617

41 He 0.047417

42 He 0.011373

43 He 0.010421

44 He 0.061652

45 He 0.077713

46 He 0.104386

47 He 0.226274

48 He 0.004333

49 He 0.026386

50 H 0.067693

51 H 0.609039

52 H 0.163420

53 H 0.148879

54 H -0.097392

55 H 0.639771

56 H 0.187646

57 H 0.450298

58 H 0.305738

59 H 0.511907

60 H 0.379577

|  |
| --- |

**Figure S3**. Snapshot of the formation of the short-lived species He_2_H^+^ during the dynamics with 30 H and 30 He atoms with the aid of the *AINR* approach.

**Table S3.** NPA charge analysis during the formation of He_2_H^+^ at the M06-2X/6-311++g level of theory for *AINR* dynamics with 30 atoms of H and 30 atoms of He taking six overall positive charge of the system (as shown in Figure S3).

1 H 0.245513

2 H 0.018273

3 He 0.008328

4 He -0.004416

5 He 0.025163

6 He 0.001936

7 He 0.020982

8 He 0.007444

9 He 0.089527

10 He 0.017899

11 He 0.014499

12 He 0.003736

13 He -0.003313

14 He 0.017335

15 He 0.033864

16 He 0.011194

17 He 0.000242

18 He 0.069747

19 He 0.023694

20 He 0.110319

21 He 0.230824

22 He 0.144853

23 H 0.026726

24 H -0.048661

25 H -0.233835

26 H 0.072088

27 H 0.204520

28 H -0.116760

29 H 0.742727

30 H -0.046207

31 H -0.073053

32 H 0.190755

33 H 0.102390

34 H 0.266926

35 H -0.190561

36 H 0.045640

37 H 0.143667

38 H 0.010809

39 H -0.249398

40 He 0.012472

41 He 0.046178

42 He 0.010882

43 He 0.020900

44 He 0.049469

45 He 0.118709

46 He 0.115546

47 He 0.186422

48 He 0.000516

49 He 0.021876

50 H 0.034288

51 H 0.709938

52 H 0.270703

53 H 0.337798

54 H 0.083405

55 H 0.629878

56 H 0.296821

57 H 0.346475

58 H 0.133768

59 H 0.511086

60 H 0.127454

|  |
| --- |

**Figure S4**. Snapshot of the formation of the short-lived species He_3_^2+^ during the dynamics with 30 H and 30 He atoms with the aid of the *AINR* approach.

**Table S4.** NPA charge analysis during the formation of He_3_^2+^ at the M06-2X/6-311++g level of theory for *AINR* dynamics with 30 atoms of H and 30 atoms of He taking six overall positive charge of the system (as shown in Figure S4).

1 H 0.637025

2 H 0.465526

3 He 0.003075

4 He 0.000838

5 He -0.008773

6 He 0.007136

7 He 0.002613

8 He 0.001454

9 He -0.001998

10 He -0.002360

11 He 0.026260

12 He 0.002658

13 He -0.001035

14 He 0.001795

15 He -0.002892

16 He 0.000852

17 He 0.018277

18 He -0.004776

19 He 0.005741

20 He 0.002386

21 He -0.004047

22 He 0.003924

23 H -0.041904

24 H 0.665233

25 H -0.089872

26 H 0.052365

27 H 0.006242

28 H -0.028295

29 H 0.562131

30 H 0.148508

31 H 0.095407

32 H -0.028660

33 H 0.035847

34 H 0.073664

35 H 0.254779

36 H 0.032318

37 H 0.032233

38 H -0.009373

39 H -0.053152

40 He 0.006423

41 He 0.001281

42 He 0.001403

43 He 0.004939

44 He -0.006117

45 He -0.001092

46 He 0.004596

47 He 0.003905

48 He 0.007654

49 He 0.013529

50 H 0.008623

51 H 0.558227

52 H 0.168203

53 H 0.098141

54 H 0.595997

55 H 0.068748

56 H 0.299496

57 H 0.662370

58 H -0.036932

59 H 0.613711

60 H 0.065746

| 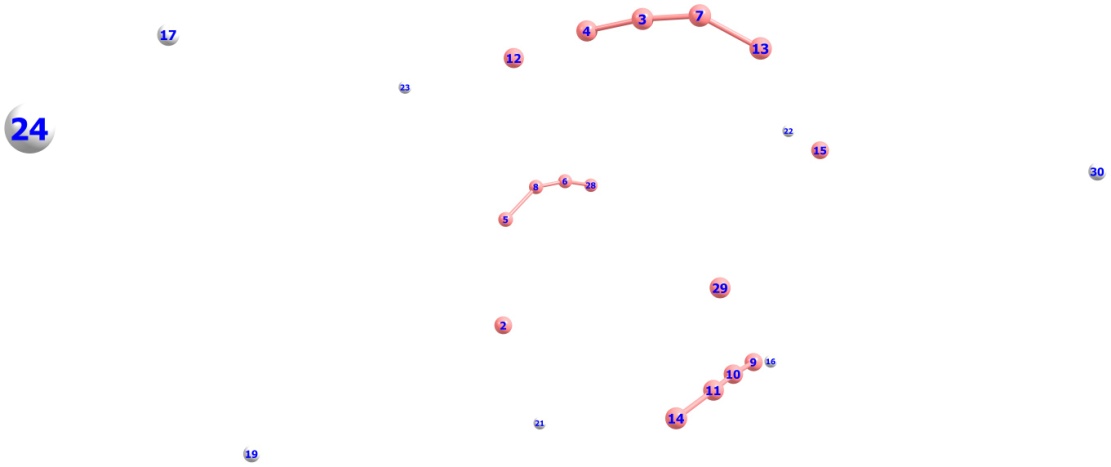 |
| --- |

**Figure S5.** Dicationic He chain formation during the *AINR* simulation of 15 H and 15 He with overall 20 positive charge.

| 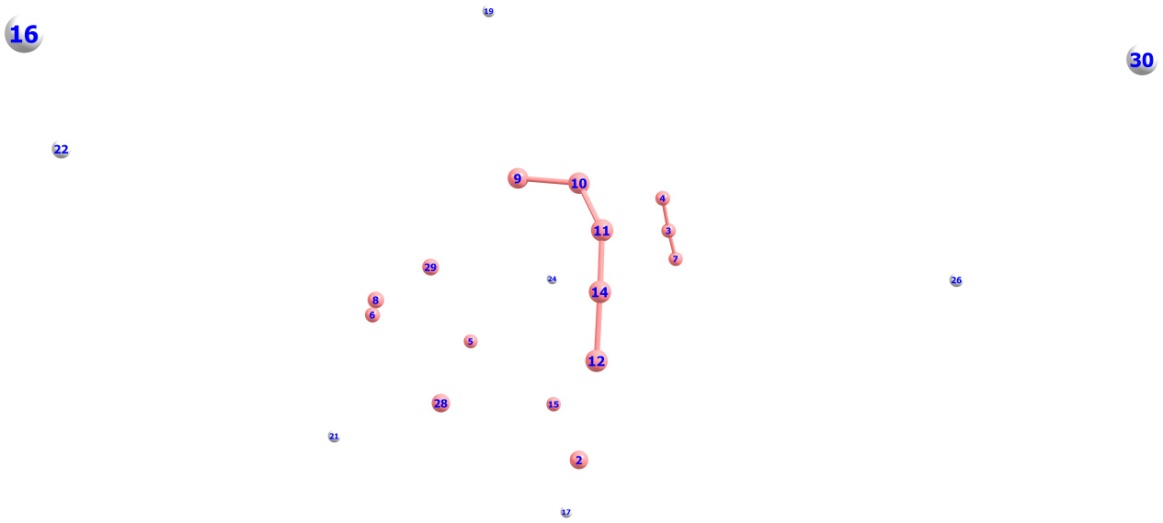 |
| --- |

**Figure S6**. Formation of dicationic He chain during the dynamics with 15 H and 15 He atoms taking twenty overall positive charge with the aid of the *AINR* approach.

**Table S5**. NPA charge analysis at the M06-2X/6-311++g level of theory for *AINR* dynamics with 15 atoms of H and 15 atoms of He taking twenty overall positive charge of the system (as shown in Figure S6).

1 H 1.000000

2 He 0.385358

3 He 0.421258

4 He 0.384395

5 He 0.454114

6 He 0.359904

7 He 0.469832

8 He 0.462498

9 He 0.441011

10 He 0.386535

11 He 0.404493

12 He 0.166085

13 He 0.381173

14 He 0.348851

15 He 0.293377

16 H 1.000000

17 H 0.999999

18 H 1.000000

19 H 1.000000

20 H 1.000000

21 H 1.000000

22 H 1.000000

23 H 1.000000

24 H 0.999998

25 H 1.000000

26 H 1.000000

27 H 1.000000

28 He 0.312395

29 He 0.328726

1. H 0.999999

| 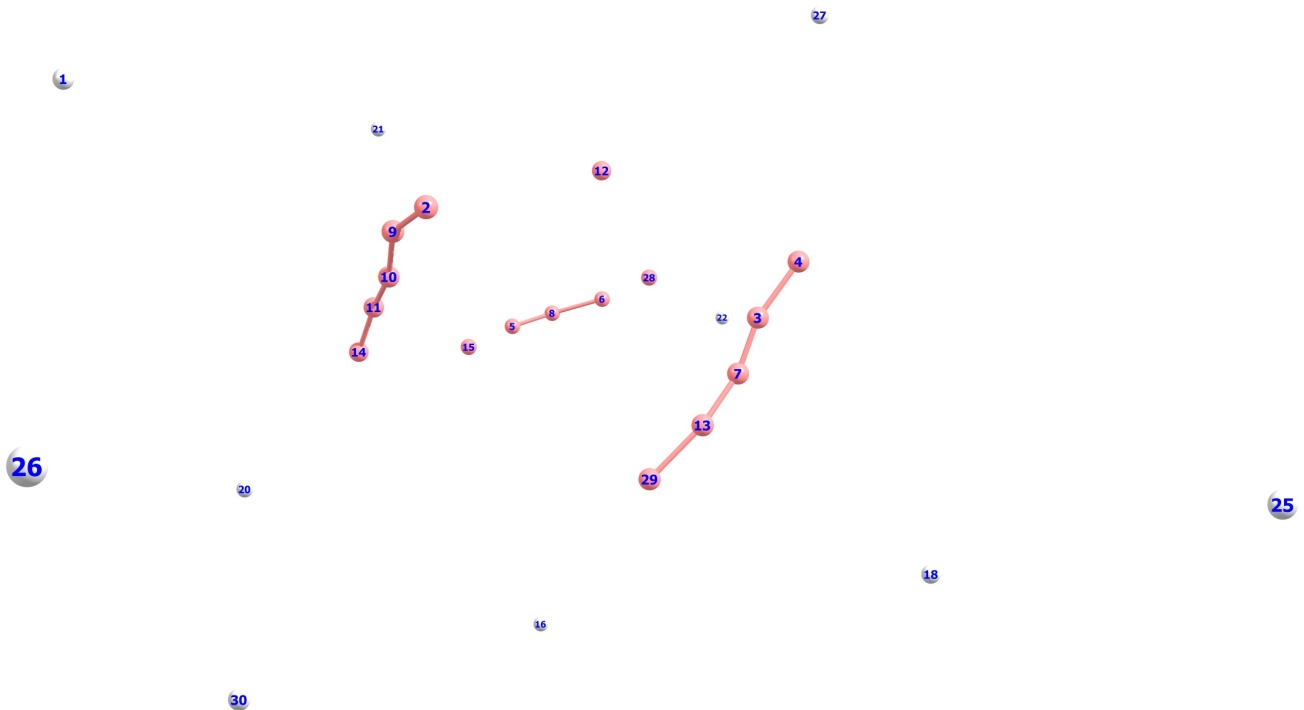 |
| --- |

**Figure S7**. Formation of the dicationic He_5_ chain during the dynamics with 15 H and 15 He atoms taking twenty overall positive charge with the aid of the *AINR* approach.

**Table S6**. NPA charge analysis at the M06-2X/6-311++g level of theory for AINR dynamics with 15 atoms of H and 15 atoms of He taking 20 overall positive charge of the system (as shown in Figure S7).

1 H 0.999989

2 He 0.225310

3 He 0.488324

4 He 0.360809

5 He 0.305277

6 He 0.544597

7 He 0.501002

8 He 0.527531

9 He 0.374922

10 He 0.436725

11 He 0.507385

12 He 0.247707

13 He 0.335169

14 He 0.423194

15 He 0.259990

16 H 1.000000

17 H 0.999998

18 H 0.999997

19 H 1.000000

20 H 0.999996

21 H 1.000000

22 H 1.000000

23 H 0.999999

24 H 1.000000

25 H 0.999999

1. H 1.000000

27 H 1.000000

28 He 0.437962

29 He 0.024119

30 H 1.000000

**The Cartesian (x, y, z) Coordinates of all the starting *AINR* dynamics geometries at the HF/6-311g Level of Theory, Employing TeraChem.**

30 H and 30 He atoms with overall (2,4,6,8…) positive charge system.

H 1.49750407 -2.48752076 0.00000000

H -6.23946262 3.21977181 -0.02000596

He 0.59858062 3.08610145 0.06657794

He -0.99326864 1.38158250 0.10007193

He -4.19364634 1.70429999 0.21394720

He -3.95043440 3.85393474 0.37654842

He -3.70447817 -0.41561367 -0.01764915

He -0.31380067 -1.49512914 -0.15135382

He 0.56881164 -0.09983126 -0.03350368

He 2.85791196 -1.25713681 -0.16279232

He 3.11946658 0.92246739 0.03296514

He 5.90267773 0.07961286 -0.07284603

He -0.14542721 -3.75599624 -0.35899987

He -3.59861334 -3.95104608 -0.34071228

He -4.87333087 -1.90045146 -0.14066510

He 4.19139277 -3.52532635 -0.38326849

He 4.49607626 1.70368399 0.08973616

He 5.59365261 3.05835041 0.20164201

He -1.66470347 3.99038057 0.36229293

He -6.67881894 1.13028429 0.15418074

He -7.12106133 -2.36952181 -0.15991601

He 6.29906743 -2.32182525 -0.29567373

H -1.77579694 -0.48293815 -0.04222174

H 0.81800177 1.28506584 0.08295957

H 2.92294723 3.30421179 0.37220645

H -2.80801727 2.75090062 0.28652590

H -5.42004342 0.05069433 -0.03903788

H -2.65807802 -2.29826214 -0.30387328

H -5.77318688 -3.26043200 -0.42785643

H 2.49261360 -3.97296352 -0.48107442

H 4.75411593 -1.75617827 -0.21336916

H 4.29852233 -0.08600767 -0.01955808

H 6.43196364 1.82761912 0.29983359

H 2.12182019 2.33745187 0.28675469

H 1.04372066 4.30597867 0.55583171

H -2.81406055 1.06841045 -0.17752376

H -1.54854516 -3.04546270 -1.45371729

H 1.81112929 -0.19746301 0.30886325

H -1.09511746 2.75307967 0.80938841

He -0.28954783 4.66704965 1.88649161

He 3.64003554 4.41964353 1.39433531

He 7.49367084 -0.50841309 -1.03105521

He 0.76242231 -4.71990709 -2.11226712

He -2.02835835 -4.73589447 -1.84286631

He -4.57286721 4.46422674 2.22515703

He -1.90280337 5.12429057 2.23486879

He 1.84209171 5.43067232 1.99155768

He -4.75597955 -4.77961158 -1.59121522

He 8.11455353 1.42075270 -0.29221885

H -5.04020382 2.61965252 0.66511906

H -3.18706882 4.81956496 1.42594089

H 0.01494911 5.80616620 1.82945907

H 5.31238137 4.53165015 1.55611426

H 4.30414543 3.31235095 1.13492526

H 3.29436243 5.73016069 1.89104145

H 7.51107895 2.72277326 1.02835756

H 5.78063377 -3.48076310 -1.02522802

H 3.04377413 -5.20555972 -1.65547808

H -0.65756926 -5.60151573 -1.89184161

H 6.76758133 4.04932082 0.75692668

29 H and 30 He atoms with overall (1,3,5,7…) positive charge system

He 1.497504 -2.487521 0.000000

He -0.379535 -1.102894 -0.004854

He 0.888836 0.641264 0.036028

He -0.229446 1.855234 0.160416

He -0.470359 4.113791 0.314915

He 0.307374 5.274526 2.010523

He -0.571138 -3.796522 -0.186706

He 0.849691 -4.252978 -1.750358

He -0.663981 -5.005673 -2.024726

He 3.079980 -4.618307 -1.924681

He 4.682595 -3.280852 -1.420403

He -2.842288 -4.078970 -0.112618

He -3.407692 -4.842776 -1.922080

He -3.468817 -2.005917 0.015564

He -3.367916 0.173160 0.184083

He -4.783623 1.423163 0.323212

He -7.077115 1.475489 0.422456

He -3.899373 3.676270 0.430317

He -5.143728 4.314186 1.708760

He -2.446982 4.774735 1.852319

He -6.013356 -1.892563 0.162603

He 2.930641 2.199109 0.050271

He 3.579500 0.099030 -0.111152

He 5.072923 -0.419740 -0.206770

He 6.395178 -1.554471 -0.334620

He 6.163464 1.437122 -0.133630

He 7.652957 2.331011 0.748170

He 8.589764 0.527642 0.024769

He 6.121388 3.875778 0.024072

He 3.833534 4.677350 0.170993

H 1.363161 3.156306 -0.083282

H -1.484480 0.591169 0.130370

H 1.382941 -0.677774 -0.061936

H 3.810212 -2.287065 -0.393521

H -1.918534 -2.784325 -0.090780

H -4.967334 -0.596949 0.283374

H -2.674195 2.221981 0.389814

H -5.906380 2.603917 0.618180

H 2.086082 4.810709 0.327268

H 4.704095 3.036252 0.015004

H 4.555224 1.306377 -0.126303

H 6.990114 -0.194835 -0.491295

H 2.849254 -1.480357 -0.295985

H 2.140711 -3.618891 -0.481566

H -2.215882 -1.118993 0.338644

H -1.680056 3.189909 1.478996

H 2.079378 0.954045 -0.357515

H -0.253791 -2.488910 -0.681356

H -4.149045 -3.072110 -0.382693

H -1.952817 -4.917838 -1.171645

H 1.359312 -5.315617 -1.682231

H 6.340394 -3.090883 -1.646396

H 5.141651 -2.064470 -1.210367

H 4.565168 -4.645502 -1.876036

H 8.188284 -0.898155 -1.243803

H 5.425844 4.940486 0.749865

H 2.443685 6.154742 1.453319

H -1.256870 5.876483 1.828902

H 7.710175 -2.329963 -0.915883

15 H and 15 He atoms with overall (20, 22, 24,26…) positive charge system

H 1.49750000 -2.48750000 0.00000000

He 0.59860000 3.08610000 0.06660000

He -0.99330000 1.38160000 0.10010000

He -4.19360000 1.70430000 0.21390000

He -3.95040000 3.85390000 0.37650000

He -3.70450000 -0.41560000 -0.01760000

He -0.31380000 -1.49510000 -0.15140000

He 0.56880000 -0.09980000 -0.03350000

He 2.85790000 -1.25710000 -0.16280000

He 3.11950000 0.92250000 0.03300000

He -0.14540000 -3.75600000 -0.35900000

He -3.59860000 -3.95100000 -0.34070000

He -4.87330000 -1.90050000 -0.14070000

He 4.16285497 -2.80047683 -0.12061225

He -1.66470000 3.99040000 0.36230000

H -1.77580000 -0.48290000 -0.04220000

H 0.81800000 1.28510000 0.08300000

H -2.80800000 2.75090000 0.28650000

H -5.42000000 0.05070000 -0.03900000

H -2.65810000 -2.29830000 -0.30390000

H -5.77320000 -3.26040000 -0.42790000

H 2.49260000 -3.97300000 -0.48110000

H 4.29850000 -0.08600000 -0.01960000

H -2.81410000 1.06840000 -0.17750000

H -1.54850000 -3.04550000 -1.45370000

H 1.81110000 -0.19750000 0.30890000

H -1.09510000 2.75310000 0.80940000

He 0.76240000 -4.71990000 -2.11230000

He -2.02840000 -4.73590000 -1.84290000

H -0.65760000 -5.60150000 -1.89180000
